# Video Clinics for Nasal Bone Injuries in COVID Times - Is it a Valid Tool for Routine Practice?

**DOI:** 10.1055/s-0044-1801313

**Published:** 2025-04-15

**Authors:** Ved Narang, Megan McGlone, Nick Calder

**Affiliations:** 1 Department of Ear, Nose, Throat, Head, and Neck Surgery, University Hospital, Monklands, Airdrie, Scotland; 2 University of Glasgow Medical School, Wolfson Medical School Building, Glasgow, United Kingdom

**Keywords:** nasal bones, video conferencing, outpatient clinics

## Abstract

**Introduction** We previously investigated the impact of video clinics on the management of closed nasal bone injuries during the coronavirus disease 2019 (COVID-19) pandemic. The aim of that study was to assess the feasibility of managing patients at their homes or workplaces, with instructions to attend outpatient clinics the next working day if they suspected any deviation or reduced nasal airflow, for further evaluation.

**Objective** To compare the results of our previous research with the traditional, in-person nasal injury clinics, using the same criteria as in our prior investigation.

**Methods** We analyzed 3 months of preexisting data from video clinics and collected 6 months of data from face-to-face clinics. We gathered information on the number of patients, categorized by age and gender, as well as records of missed appointments (DNAs) and the requirement for nasal manipulations. Data analysis was conducted using the Chi-Squared test in SciPy Python 3.0 (open source).

**Results** The statistical analysis revealed no significant differences between the 2 groups concerning the number of patients with closed nasal bone injuries, both under and over 18 years of age ( *p* -value: 0.961), the rate of missed appointments ( *p* -value: 0.0734), and the need for fracture reduction ( *p* -value: 0.0734).

**Conclusions** The findings suggest that video clinics are equally effective in managing suspected nasal bone injuries and should not be restricted to emergency situations. However, it is advisable to adopt a balanced approach considering the additional costs associated with appointments.

## Introduction and Objective

The coronavirus disease 2019 (COVID-19) pandemic compelled doctors to develop an innovative tool for reducing human interaction and managing patients with suspected nasal bone injuries (NBIs) through video clinics (VCs). 1 The authors sought to assess the validity of their previous findings by comparing them with those of another study that did not utilize VCs. For the purposes of this study, the standard in-person clinics were referred to as emergency clinics (ECs) and were employed when isolation measures were relaxed. During the initial follow-up visit, patients were examined in person at ECs.

The primary objective of the present study was to compare the number of patients requiring nasal bone manipulation (NBM) when attending VCs with those attending ECs. Our secondary objective was to analyze the outcomes based on age, gender, and non-attendance (did not attend, DNA) rates. Our goal was to determine whether these two types of clinics could effectively serve as substitutes for one another without compromising patient outcomes.

## Methods

Ethical considerations for the present study encompassed the following aspects: We retrospectively gathered data for our current observational study from the Trak Care system within the National Health Service (NHS), while the outcomes were extracted from each patient's individual file. As the present study constituted a part of the audit process, a separate approval from the NHS quality department was not deemed necessary. A previous prospective observational study involving video clinics (VCs) had received approval under project ID 13183, dated 09/02/2021.

All patients presenting at the accident and emergency (A&E) departments of NHS trust hospitals with suspected nasal bone injuries (NBIs) were offered symptomatic treatment. Radiological investigations were not requested for patients with closed NBIs. The attending physicians scheduled appointments at a dedicated emergency clinic (EC) 7 to 14 days after the initial visit. Patients with septal hematoma or compound NBIs were managed by the on-call ear, nose, and throat (ENT) team and were excluded from the study group.

At the EC, patients underwent assessment through visual inspection of the external nose, followed by an internal examination of the nasal cavity using the Thudicum speculum. If necessary, nasal endoscopy was performed. If clinically indicated, patients underwent nasal bone manipulation (NBM) either immediately or were scheduled later under general anesthesia (GA). For those undergoing NBM under local anesthesia (LA), Lignospan special (lidocaine 2% + 1.25% adrenaline) was injected alongside the nasal bones. A few patients chose NBM without LA injection, and while we respected their preference, we did not maintain a record of their numbers. Deformities were corrected until continuity was restored, and symmetry was achieved. Each patient was asked to assess their nose in a looking glass after correction of the deformity, and if satisfied with the shape of their nose and nasal breathing, they were discharged.

For clarity throughout this article, we will refer to patients from the previous study as the VC group and those from the present study as the EC group. The key distinction between the VC and EC groups lies in the additional step of attending a VC between the A&E visit and the EC, in the VC group. The VC allowed us to evaluate patients via a video call, after which they were either instructed to attend the EC on the next working day or were discharged. 1

Preexisting 3-month data starting in July 2020 were available for the VC group. 1 Nearly identical data, apart from the VC appointment, were collected for the EC group over a 6-month period starting in December 2020. Data were compiled for age, gender, DNA rates, and the number of patients requiring NBM. Statistical analysis was conducted to assess the significance of the data using a Chi-squared test in SciPy Python 3.0 (open source).

## Results

The EC group consisted of 134 patients, compared to 42 patients in the VC group. In the EC group, there were 89 males and 45 females, whereas the VC group had 23 males and 19 females. The male-to-female ratio was 1.2:1 during the peak of COVID-19, compared to 1.97:1 when isolation rules were relaxed. The difference between the 2 groups was not statistically significant ( *p* -value: 0.1706, with 1 degree of freedom [df]).

In the VC group, there were 13 patients under the age of 18, and 29 patients older than 18, resulting in a ratio of 1:2.23. In the EC group, there were 42 patients under the age of 18, and 92 older than 18, with a ratio of 1:2.19. The difference between the 2 groups was not statistically significant ( *p* -value: 0.961, with 1 df).

Some patients DNA their follow-up appointments, totaling 26.19% in the VC group compared to 20.14% in the EC group. The difference was not statistically significant ( *p* -value: 0.073, with 1 df; Table 1 ).

**Table 1:** The total number of patients missing appointments after one to two weeks of initial examination in A&E

	Video clinics	Emergency clinic
Patients attending A&E	42	134
Patients attending clinic appointment	31	107
Total number of DNA patients	11*	27

**Abbreviation:** A&E, accident and emergency.

**Note:** This table shows the total number of DNA patients, including those who could not attend for other* reasons ( *p* -value: 0.073).

- Out of 11 patients, 5 did not attend VC but attended the EC as direct appointments. They failed to attend VC because of technical failure to connect their devices through the internet or due to scheduling difficulty caused by intervening holidays because VC was limited to one day a week, leaving six patients as the actual DNA number.

Excluding patients who DNA the VC group due to technical issues, 6 (14.28%) patients were lost to follow-up (that is, DNA) in the VC group compared to 27 (20.14%) in the EC group. The difference between the 2 groups was not significant ( *p* -value: 0.39; Table 2 ).

**Table 2:** Number of patients missing appointments for other reasons during video clinics versus their own decision during emergency clinics

	Video clinics	Emergency clinics
Number attending clinic appointment	36	107
DNA patients, excluding patients who could not attend for other* reasons.	6*	27
Total	42	134

**Abbreviation:** DNA, did not attend.

**Note:** This table shows the number of DNA patients, excluding those who could not attend for technical reasons ( *p* -value: 0.39).

Five out of 11 patients could not attend due to technical reasons, and their numbers have been subtracted from the DNA in the previous table, leaving 6 as the final DNA number in the VC group.

Twelve (28.57%) patients needed NBM in the VC group, compared to 44 (32.83%) in the EC group. The difference between the 2 groups was not significant ( *p* -value: 0.7430, with 1 df; Table 3 ).

**Table 3:** Comparison of patients needing nasal bone manipulations under local or general anesthesia in two clinic groups

	Video clinics	Emergency clinics
Patients needing nasal bone manipulation	12	44
Patients not needing nasal bone manipulation	30	90
Total	42	134

Two patients with a preexisting septum deformity were scheduled for septoplasty later in the VC group, compared to six patients in the EC group. Two patients in each group requested NBM under GA ( Table 4 ).

**Table 4:** Final analysis of the patients in two clinic groups

	Video clinics	Emergency clinics
Total patients	42	134
Discharged from the emergency clinic (NBM not required)	24*	63
DNA (lost to follow-up)	6	27
Patients needing NBM	12	44
Under LA	10	42
Under GA	2	2
Preexisting deformity for septoplasty later	2	6

**Abbreviations:** GA, general anesthesia; LA, local anesthesia; NBM, nasal bone manipulation.

**Note:** Total patients discharged—24 (13 [after video clinic, VC] + 11 [from the emergency clinic following VC]). Data from the previous study. 1

## Discussion

Close human interaction played a significant role in the widespread transmission of COVID-19 during the pandemic. 2 The primary objective of our initial study was to reduce human interaction by implementing VCs, thereby mitigating the risk of cross infections and potential COVID virus transmission. 1

Our literature search using the keywords *video clinics* and *nasal bone injuries* did not yield similar studies for comparison with our results. The absence of comparable studies might be attributed to the lack of forced isolation periods before the COVID-19 pandemic and the absence of video technology for patient examinations prior to the pandemic. We believe that our study represents a pioneering effort in this regard.

Closed NBIs are typically uncomplicated and do not necessitate radiological investigations. 3 Therefore, we did not request X-rays for any patients with closed NBIs. Dedicated clinics for NBIs have demonstrated superior outcomes compared to general ear, nose, and throat (ENT) clinics. 4 In alignment with this practice, we advised patients to attend a dedicated EC within 2 weeks of their nasal injury. During a 6-month period, the number of patients increased from 42 in the 3-month video clinic (VC) period to 134 attending the EC, marking a 1.59-fold increase when time periods were normalized. The rise in the number of patients could be attributed to the relaxation of isolation rules, leading to increased social interaction and, consequently, more nasal injuries.

Historically, large study groups have reported a male-to-female ratio ranging from 2:1 to 6.8:1. 5 6 7 8 In comparison, we observed a ratio of 1.2:1 during the peak of COVID-19 (VC group) 1 and 1.97:1 when isolation rules were relaxed (EC group) in our study cohorts. The gender ratio remained statistically insignificant between the two groups, indicating that men are consistently more prone to nasal injuries. This tendency in men persisted whether they were constrained to stay at home during COVID-19 or allowed to venture out when restrictions were lifted. The higher incidence of nasal injuries in men may be attributed to their more aggressive social behaviors. 9

Previous research has identified the highest incidence of NBIs among individuals aged 15 to 40 years, with those in their 20s (31.8%) being the most commonly affected, followed by individuals in their teens (22.3%), 30s (19.7%), and 40s (16.1%). 10 To maintain clinical relevance, we categorized patients into 2 age groups: above and below 18 years of age, as further subgroups may not be clinically meaningful. When time periods were equalized, the difference in patient numbers between the 2 age groups was statistically insignificant ( *p* -value: 0.961). The higher number of patients older than 18 may be attributed to their greater representation in the overall UK population. 11 Additionally, this could be influenced by social factors, as individuals over 18 often lead more independent lives and venture out more frequently, increasing their risk of nasal injuries.

Closed fracture reduction has proven effective in managing patients with external nasal deviation. 12 Nasal bone manipulation was performed within 7 to 14 days of the injury date under LA for all patients, whether adults or children, during their initial EC appointment. 13 Some authors have recommended a shorter interval of 2 to 3 days for pediatric patients. 14 Furthermore, there are suggestions of a maximum of 7 days for children and 10 days for adults. 15 16 However, for simplicity and flexibility in scheduling, we chose to manage cases between 7 and 14 days postinjury.

Allowing 1 to 2 weeks from the time of injury enabled localized edema to subside and offered a better assessment of the external nose. Overall, 28.75% of patients in the VC group required NBM, compared with 32.83% in the EC group. The difference in the number of patients requiring NBM between the 2 groups was statistically insignificant ( *p* -value: 0.743). This suggests that no patients requiring NBM were overlooked during the VC period, even when COVID-19 restrictions were at their peak.

For patients with multiple nasal bone fracture segments, a duration greater than 2 weeks has been recommended for NBM. 17 However, confirming the presence of multiple fracture segments requires radiological investigation, which is not standard practice at our center. The lack of knowledge about multiple fracture segments does not affect the management of NBIs, as the primary goal is to restore symmetry through digital manipulation.

Statistical analysis did not reveal any significant difference in the rates of DNA when comparing patients who failed to attend follow-up clinics between the two groups ( *p* -value: 0.39). This insignificance persisted when technical reasons for the inability to attend video calls were considered in the VC group ( *p* -value: 0.073). The lack of statistical significance in DNA rates suggests that the use of video consultation did not result in an increase in DNA numbers. Some patients with closed NBIs may choose to discharge themselves from follow-up, regardless of whether they belonged to the VC or EC group.

Emergency clinics have long been a proven method for managing closed NBIs. They were the standard practice before isolation guidelines were enforced during COVID-19, and they remained so after the guidelines were relaxed. Emergency clinics offer essential advantages, including direct human interaction, tactile examination of injured nasal bones, and real-time assessment of the nasal cavity, aspects that were not possible with VCs. However, the limitations of VCs in these areas were partly mitigated by using alternative strategies to assess nasal shape and ensure good and approximately equal airflow from both sides. 1

The benefits of VCs include reducing unnecessary hospital visits, enabling examinations from the comfort of patients' homes or offices, and proving particularly valuable during the pandemic. 1 Video assessments, however, require an additional appointment between the initial assessment in the accident and emergency (A&E) department and the EC visit. A substantial proportion of patients (41.9%) were discharged after attending the VC from their homes, 1 saving them time and money. Only those with a definite need for NBM or a suspicion of requiring it were called to attend the EC on the next working day.

The inclusion of an extra appointment slot for video clinics may not align with hospital management goals, as they often grapple with managing growing waiting lists. However, if the addition of this appointment is feasible, it can be integrated into routine clinic schedules. Video clinics were associated with high patient satisfaction rates, 1 and no adverse outcomes were reported. Therefore, we recommend their use for patients residing in remote areas, during adverse weather conditions, or in situations of forced isolation.

## Conclusions

The video clinic (VC) proved to be just as effective in managing patients with suspected nasal bone injuries (NBIs) when compared to the traditional, in-person, external clinic (EC). We found no statistical differences in outcomes when considering variables such as gender, age groups, rates of missed appointments (DNAs), and the percentage of people requiring NBMs).

Therefore, incorporating VC into routine clinical practice, albeit at the cost of an additional appointment, is a viable option. A balanced approach to the use of VCs could yield significant benefits for both patients and administrators.

## References

[1] Narang V Eswarappa V Calder N Impact of video clinics in the management of fracture nasal bones in COVID times Rhinol Online 2021 4 127 130

[2] Laxminarayan R Malani A Economics of infectious diseases Oxford University Press 2011

[3] Sharp J F Denholm S Routine X-rays in nasal trauma: the influence of audit on clinical practice J R Soc Med 1994 87 03 153 154 8158594 10.1177/014107689408700313 PMC1294397

[4] Pinto R Wright R Ghosh S Nasal fractures: a dedicated clinic providing reduction under local anaesthesia improves time to manipulation Ann R Coll Surg Engl 2020 102 06 418 421 32326744 10.1308/rcsann.2019.0185 PMC7388945

[5] Renkonen S Vehmanen S Mäkitie A Blomgren K Nasal bone fractures are successfully managed under local anaesthesia - experience on 483 patients Clin Otolaryngol 2016 41 01 79 82 25943694 10.1111/coa.12456

[6] Nishioka H Kondoh S Yuzuriha S Convex bone deformity after closed reduction of nasal bone fracture J Plast Reconstr Aesthet Surg 2018 71 01 85 89 28918998 10.1016/j.bjps.2017.08.022

[7] Turvey T A Midfacial fractures: a retrospective analysis of 593 cases J Oral Surg 1977 35 11 887 891 269930

[8] Kang B H Kang H S Han J J A retrospective clinical investigation for the effectiveness of closed reduction on nasal bone fracture Maxillofac Plast Reconstr Surg 2019 41 01 53 31824891 10.1186/s40902-019-0236-y PMC6879701

[9] Stanaland A Gaither S “Be a Man”: The Role of Social Pressure in Eliciting Men's Aggressive Cognition Pers Soc Psychol Bull 2021 47 11 1596 1611 33504285 10.1177/0146167220984298

[10] Hwang K You S H Kim S G Lee S I Analysis of nasal bone fractures; a six-year study of 503 patients J Craniofac Surg 2006 17 02 261 264 10.1097/00001665-200603000-00010[Internet] 16633172

[11] Gov.uk. [cited 2023 Oct 31]. Available from:https://www.ons.gov.uk/releases/ethnicgroupbyageandsexenglandandwalescensus2021

[12] Park H-K Lee J-Y Song J-M Kim T-S Shin S-H The retrospective study of closed reduction of nasal bone fracture Maxillofac Plast Reconstr Surg 2014 36 06 266 272 10.14402/jkamprs.2014.36.6.266[Internet] 27489845 PMC4283532

[13] Park C H The current knowledge of the treatment of nasal bone fractures J Rhinol 2011 18 94 101

[14] Goode R L Spooner T R Management of nasal fractures in children. A review of current practices Clin Pediatr (Phila) 1972 11 09 526 529 10.1177/000992287201100915[Internet] 5073805

[15] Harrison D H Nasal injuries: their pathogenesis and treatment Br J Plast Surg 1979 32 01 57 64 10.1016/0007-1226(79)90063-8[Internet] 427309

[16] Rohrich R J Adams W P Jr Nasal fracture management: minimizing secondary nasal deformities Plast Reconstr Surg 2000 106 02 266 273 10946923 10.1097/00006534-200008000-00003

[17] Yoon H Y Han D G Delayed reduction of nasal bone fractures Arch Craniofac Surg 2016 17 02 51 55 10.7181/acfs.2016.17.2.51[Internet] 28913255 PMC5556871

